# Transverse Electric Inverse Scattering of Conductors Using Artificial Intelligence

**DOI:** 10.3390/s25123774

**Published:** 2025-06-17

**Authors:** Chien-Ching Chiu, Po-Hsiang Chen, Yen-Chen Chang, Hao Jiang

**Affiliations:** 1 Department of Electrical and Computer Engineering, Tamkang University, Tamsui 251301, Taiwan; 810440031@gms.tku.edu.tw (P.-H.C.); 411490120@o365.tku.edu.tw (Y.-C.C.); 2 School of Engineering, San Francisco State University, San Francisco, CA 94132, USA; jianghao@sfsu.edu

**Keywords:** inverse scattering, conductor, sensing electrical field, Direct Sampling Method (DSM), U-Net, Transverse Electric (TE), electromagnetic imaging

## Abstract

Sensors are devices that can detect changes in the external environment and convert them into signals. They are widely used in fields like industrial automation, smart homes, medical devices, automotive electronics, and the Internet of Things (IoT), enabling real-time data collection to enhance system intelligence and efficiency. With advancements in technology, sensors are evolving toward miniaturization, high sensitivity, and multifunctional integration. This paper employs the Direct Sampling Method (DSM) and neural networks to reconstruct the shape of perfect electric conductors from the sensed electromagnetic field. Transverse electric (TE) electromagnetic waves are transmitted to illuminate the conductor. The scattered fields in the x- and y-directions are measured by sensors and used in the method of moments for forward scattering calculations, followed by the DSM for initial shape reconstruction. The preliminary shape data obtained from the DSM are then fed into a U-net for further training. Since the training parameters of deep learning significantly affect the reconstruction results, extensive tests are conducted to determine optimal parameters. Finally, the trained neural network model is used to reconstruct TE images based on the scattered fields in the x- and y-directions. Owing to the intrinsic strong nonlinearity in TE waves, different regularization factors are applied to improve imaging quality and reduce reconstruction errors after integrating the neural network. Numerical results show that compared to using the DSM alone, combining the DSM with a neural network enables the generation of high-resolution images with enhanced efficiency and superior generalization capability. In addition, the error rate has decreased to below 15%.

## 1. Introduction

Electromagnetic imaging relies fundamentally on the precise detection and characterization of electromagnetic fields scattered by objects of interest. Among various detection mechanisms, magnetic sensors and systems offer distinct advantages due to their high sensitivity to weak magnetic fields, wide dynamic range, and compatibility with different environmental conditions. In particular, magnetic sensors enable the non-invasive acquisition of field data with high spatial and temporal resolution, which is critical for accurately reconstructing the target’s properties in electromagnetic imaging. Techniques such as magnetic induction tomography, magnetic resonance imaging, and magnetoacoustic imaging exemplify the integration of magnetic sensing technologies into electromagnetic imaging frameworks. Furthermore, the development of advanced magnetic sensor systems, including superconducting quantum interference devices, fluxgate magnetometers, and giant magnetoresistance sensors, has significantly expanded the capabilities of electromagnetic imaging in both scientific research and industrial applications. These advantages have led to the widespread integration of magnetic sensors into various imaging modalities, including biomedical imaging and industrial non-destructive evaluation [1]. Thus, magnetic sensors and systems not only enhance the performance of existing electromagnetic imaging methods but also enable the creation of novel imaging modalities, addressing key challenges such as low signal-to-noise ratios, high computational demands, and imaging in complex environments.

Electromagnetic imaging is a technique in which electromagnetic waves are transmitted to illuminate and analyze an unknown scatterer and subsequently reconstruct its shape, size, location, and material properties by analyzing the scattered field reflected from the object. Due to its effectiveness, electromagnetic imaging has been widely used in various domains, including biomedical imaging, geophysical exploration, remote sensing, and medical imaging. In particular, imaging based on transverse electric (TE) wave excitation remains less investigated than its transverse magnetic (TM) counterpart, due to the difficulty of satisfying boundary conditions associated with electric field continuity and weaker scattering behavior [2,3]. However, challenges arise due to its inherent nonlinearity and ill-posedness, and the computational complexity associated with optimization algorithms and parameter selection. In previous studies, the inverse scattering problem has often been reformulated as an optimization problem for solution. However, traditional algorithms require substantial computational resources and time, making it difficult to efficiently obtain high-resolution reconstructed images.

In parallel, non-iterative techniques such as the Direct Sampling Method (DSM) have attracted growing attention due to their computational simplicity and robustness to noise. The DSM constructs an indicator function based directly on the measured scattered fields, allowing rapid estimation of the object’s support without iterative solvers or prior information [4,5,6].

Objective function methods are typically employed to determine the extrema of a given function, such as minimizing the error between the measured and calculated scattered fields. Over the past two decades, extensive research has been conducted in this area. Two primary subtypes of electromagnetic imaging algorithms are identified: local search and global search methods. In general, solutions are obtained using either gradient-based methods or global optimization algorithms. Gradient-based methods are more suitable for local search problems, whereas global optimization algorithms are preferred for broader search domains. There are three principal categories of neural network-based learning methods: direct learning, objective function enhancement through learning, and physics-assisted learning. The direct learning approach utilizes neural networks to compute wave equations directly, and the learning-assisted objective function method utilizes neural networks during the iterative process to determine optimal iteration directions for image reconstruction. Meanwhile the physics-assisted learning method leverages Maxwell’s equations to first compute certain physical parameters and then apply neural networks to refine these parameters for more accurate object reconstruction. Currently, methods for addressing the electromagnetic imaging problems can generally be divided into two principal approaches: objective function methods [7,8,9,10,11,12,13,14,15,16,17,18] and neural network-based learning methods [19,20,21,22,23,24,25,26,27].

In the study of objective function methods for microwave imaging, in 2022, Xie et al. proposed an image-domain scattering parameter determination technique for UltraWideBand (UWB) MicroWave Photonic (MWP) signals. Particle Swarm Optimization (PSO) was employed to detect the parameters of Attributed Scattering Centers (ASCs) within the image domain. Additionally, Orthogonal Matching Pursuit (OMP) eliminated unnecessary computations. The results demonstrated superior accuracy and completeness in obtaining ASC parameters from UWB MWP signals [14]. In the same year, Zhang et al. introduced a Group Sparsity Penalized Contrast Source Inversion (GSPCSI) algorithm to address inverse scattering problems. Computational simulations indicated that this approach successfully mitigates noise interference, leading to significant improvements in imaging performance [16]. More recently, in 2024, Wang et al. introduced a new approach called the Cross-Correlated Subspace Optimization Method (CC-SOM). The method employed fast algorithms, leveraging the properties of singular value decomposition (SVD) to simplify large-scale matrices while incorporating Fast Fourier Transform (FFT) to enhance computational speed. The key advantage of this method lies in its superior computational accuracy; however, it also introduces increased computational complexity [18].

In recent years, various neural network-based approaches have been proposed to enhance microwave imaging. In 2019, Wei et al. introduced a method leveraging the Dominant Current Scheme (DCS) to obtain an initial estimation of the dielectric permittivity distribution, which was then refined using a U-Net model for accurate reconstruction. The results indicated that this approach is effective only for cases with low dielectric permittivity [19]. In 2020, Xiao et al. proposed a technique that first employed the Born Approximation (BA) to estimate an initial 3D representation of the scattering object. The initial image was then refined using the Monte Carlo method before being processed by a 3D U-Net and variational Born iteration method (VBIM) architecture for final reconstruction. Numerical simulations indicated that 3-D U-Net did better than VBIM in both precision and efficiency [20]. In 2021, Huang et al. proposed a novel loss function for U-Net, integrating Mean Squared Error (MSE) at the pixel level and the Structural Similarity Index (SSIM) to enhance reconstruction quality. Numerical results demonstrated that this method achieved high performance and superior resolution in microwave imaging applications [21]. In 2022, Chiu et al. proposed an AI-driven approach for reconstructing electromagnetic images of biaxial objects. The study combined the DCS with the Backpropagation Scheme (BPS) for initial estimations and subsequently utilized a U-Net deep learning framework for image reconstruction. Numerical simulations indicated that the DCS-based initial estimation provided superior accuracy and noise robustness compared to the BPS method [23]. Most recently, in 2024, Chiu et al. introduced an improved deep learning-based method that integrated a Modified Contrast Scheme (MCS) with a U-Net neural network for reconstructing biaxial electromagnetic images. Experimental results demonstrated that, compared to the DCS, the MCS exhibited higher accuracy and better noise resistance [26].

In recent years, most researchers have focused on electromagnetic imaging of dielectric materials or the transmission of TM waves for conductor reconstruction, while studies on TE wave transmission for conductors remain scarce. The values of this research include the following:

1. To the best of our knowledge, most of the aforementioned papers focused mainly on transmitting TM wave electromagnetic imaging. There is no publication that transmits TE waves and combines AI techniques to reconstruct conductors in free space.
2. Unlike scalar TM waves, TE-polarized waves comprise both $E_{x}$ and $E_{y}$ components, which are inherently coupled. This work explicitly models these vectorial interactions in the forward problem, addressing a computationally challenging yet underexplored aspect of TE-based imaging.
3. Traditional algorithms require a long calculation time. Furthermore, processing is not instantaneous. So, we propose training U-Net to achieve instantaneous imaging. In short, good reconstruction can be achieved by utilizing our proposed single-axis object selection method.
4. Cooperating with the DSM to estimate the initial permittivity distribution can effectively accelerate the training process.
5. Numerical simulations reveal that our introduced method successfully achieves good performance and high-resolution reconstruction of conductor shapes in TE wave electromagnetic imaging.

The proposed method is structured as a five-stage pipeline: (1) TE-polarized waves illuminate the target conductor; (2) the scattered fields in the x- and y-directions are collected and used in forward modeling; (3) the Direct Sampling Method (DSM) produces an initial estimate of the shape; (4) a U-Net is trained with DSM-derived data to learn optimal parameters; and (5) the trained model is used to reconstruct high-resolution images. Accordingly, Section 2 presents the theoretical formulations of the electromagnetic field and the DSM, corresponding to the TE illumination (Step 1) and initial shape estimation (Step 3). Section 3 introduces the U-Net architecture used for deep learning-based refinement (Step 4). Section 4 reports the results of numerical simulations validating the trained model (Step 5), based on scattered field data acquisition (Step 2). Section 5 concludes this paper.

## 2. Materials and Methods

**Figure 1:**
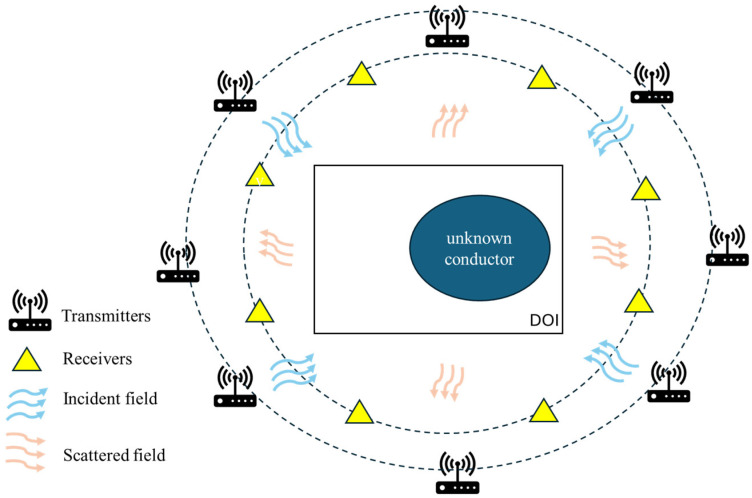
Unknown conductor in free space.

In this section, we present the theoretical formulation for electromagnetic scattering under transverse electric (TE) wave illumination [28,29]. We set a perfect conductor situated in free space, stretching to infinity along the z-axis. The cross-sectional shape in the (x, y) plane is defined by the polar equation ρ=F(θ), which is illustrated in Figure 1. The angle ϕ used in Equations (1) and (2) denotes the incident angle in the cylindrical coordinate system, and the incident field is modeled as a time-harmonic plane wave propagating in the two-dimensional xy-plane under transverse electric (TE) polarization.

Let ${\overset{⃑}{H}}^{i}$ represent the incident magnetic field and ${\overset{⃑}{E}}^{i}$ represent the incident electric field.

(1) $${\overset{⃑}{H}}^{i}\left(x,y\right)=e^{-jk_{0}\left(x\sin\phi +y\cos\phi \right)}$$

(2) $${\overset{⃑}{E}}^{i}\left(x,y\right)=\sqrt{\frac{u_{0}}{\varepsilon_{0}}}\left(-\cos\emptyset \hat{x}+\sin\phi \hat{y}\right)e^{-jk_{0}\left(x\sin\phi +y\cos\phi \right)}$$

Since TE-polarized waves and two-dimensional conductors have the characteristics outlined in Equations (3) and (4) can be derived.

(3) $$\left\{\begin{array}{c} \overset{⃑}{H}=H_{Z}\hat{z}\\ \overset{⃑}{E}=E_{x}\hat{x}+E_{y}\hat{y}\\ {\overset{⃑}{J}}_{sm}=J_{sm}\hat{z}\\ \frac{\partial }{\partial z}=0 \end{array}\right.$$

(4) $$\left({\nabla \;}^{2}+k_{0}^{2}\right)\overset{⃑}{H}=-j\omega \varepsilon_{0}{\overset{⃑}{J}}_{sm}\delta \left(\rho -F(\theta )\right)$$

(5) $$\rho =|\overset{⃑}{r}-{\overset{⃑}{r}}^{\prime }|=\sqrt{{\left(x-F(\theta^{\prime })cos\theta^{\prime }\right)}^{2}+{\left(y-F(\theta^{\prime })sin\theta^{\prime }\right)}^{2}}$$

$E_{x}$ and $E_{y}$ are the incident electric fields along the x- and y-axes. ${\bar{J}}_{sm}$ is the induced surface magnetic current density. Through equivalent formulations, the scattered magnetic field, ${\overset{⃑}{H}}^{s}=\overset{⃑}{H}-{\overset{⃑}{H}}^{i}$ at a random point (x, y) in the Cartesian coordinate system or (r, θ) in polar coordinate representation outside the scatterer, is given by

(6) $$H_{z}^{s}(\overset{⃑}{r})=\int_{c}\frac{j}{4}H_{0}^{\left(2\right)}\left(k_{0}\left|\overset{⃑}{r}-{\overset{⃑}{r}}^{\prime }\right|\right)\left(-j\omega \varepsilon_{0}\right)J_{sm}\left({\overset{⃑}{r}}^{\prime }\right)dl^{\prime },\;\overset{⃑}{r}=\left(x,y\right)$$

where $H_{0}^{\left(2\right)}$ is the Hankel function of the second kind of order zero, and $J_{sm}\left(\theta \right)$ is the induced surface magnetic current density. C is the outer contour of the conductor. To comply with the boundary condition, the total tangential electric field must be zero on the scatterer’s surface, which leads to an integral equation for $J_{sm}\left(\bar{r}\right)$.

(7) $$\hat{n}\times \left(\frac{1}{j\omega \varepsilon_{0}}\nabla \times \overset{⃑}{H}\right)=0$$

(8) $$\hat{n}=\frac{\hat{x}(F(\theta )cos\theta +F^{\prime }(\theta )sin\theta )+\hat{y}(F(\theta )sin\theta -F^{\prime }(\theta )cos\theta )}{\sqrt{F^{2}(\theta )+{F^{\prime }}^{2}(\theta )}}$$

Here $\hat{n}$ corresponds to the unit normal vector perpendicular to the conductor’s surface and pointing outward. In the direct scattering problem, the scattered fields $E^{s}$ are determined based on the assumption of a known object shape. This is accomplished by first solving $J_{sm}$ in the above Equation (7) and calculating $H_{s}$ from Equation (6). In addition, Equation (9) can be derived from Equation (7).

(9) $$-\hat{n}\times {\overset{⃑}{E}}^{\;i}=\hat{n}\times {\overset{⃑}{E}}^{\;s}$$

where

(10) $$-\hat{n}\times {\overset{⃑}{E}}^{\;i}=\left[\frac{\left(F(\theta )sin\theta -F^{\prime }(\theta )cos\theta \right)}{\sqrt{F^{2}(\theta )+{F^{\prime }}^{2}(\theta )}}E_{x}^{i}+\frac{\left(F(\theta )cos\theta +F^{\prime }(\theta )sin\theta \right)}{\sqrt{F^{2}(\theta )+{F^{\prime }}^{2}(\theta )}}E_{y}^{i}\right]\hat{z}$$

$$\hat{n}\times {\overset{⃑}{E}}^{\;s}=\frac{jk_{0}}{4}\int_{0}^{2\pi }\frac{1}{\rho }\left\{\begin{array}{c} \left(F(\theta^{\prime })cos\theta^{\prime }-F(\theta )cos\theta )(F(\theta )cos\theta +F^{\prime }(\theta )sin\theta \right)\\ +(F(\theta^{\prime })sin\theta^{\prime }-F(\theta )sin\theta )(F(\theta )sin\theta -F^{\prime }(\theta )cos\theta ) \end{array}\right\}$$

(11) $$\frac{1}{\sqrt{F^{2}(\theta )+{F^{\prime }}^{2}(\theta )}}H_{1}^{\left(2\right)}(k_{0}\rho )J_{sm}\left(\theta^{\prime }\right)\sqrt{F^{2}(\theta^{\prime })+{F^{\prime }}^{2}(\theta^{\prime })}d\theta^{\prime }$$

Here ${\overset{⃑}{E}}^{\;s}$ is the scattered electric field. Based on the method of moments (MoM), the conductor’s edge is partitioned into N small segments, where the induced magnetic current on each small segment can be regarded as a constant.

(12) $$J_{sm}\left(\overset{⃑}{r}\right)=\sum_{n=1}^{N_{d}}{\left(J_{sm}\right)}_{n}{\left(P_{c}\right)}_{n}$$

(13) $${\left(P_{c}\right)}_{n}=\left\{\begin{array}{c} 1\;,on△C_{n}\\ 0\;,otherwise \end{array}\right.$$

where the pulse function ${\left(P_{c}\right)}_{n}$ is the basis function in the expansion, and $△C_{n}$ is the ith arc segment of the object from $\theta =2\pi (i-1)/\;N_{d}$ to $\theta =2\pi /N_{d}.\;\;N_{d}$ is the number of segments for the conductor. Let the test function be

(14) $$V_{1m}=\delta \left(x-F(\theta_{m})cos\theta_{m}\right)\;\delta \left(y-F(\theta_{m})cos\theta_{m}\right)$$

Then, take the dot product of Equation (9) with $V_{m}$.

(15) $$\left\langle \hat{n}\times {\overset{⃑}{E}}^{\;s},V_{1m}\right\rangle =\sum_{n=1}^{N_{d}}{\left(G_{1}\right)}_{mn}{\left(J_{sm}\right)}_{n}$$

where

(16) $${\left(G_{1}\right)}_{mn}=\left\{\begin{array}{cc} \begin{array}{c} \frac{jk_{0}}{4}\frac{1}{\rho_{m}}\left\{\begin{array}{c} \left(F(\theta_{n})cos\theta_{n}-F(\theta_{m})cos\theta_{m})(F(\theta_{m})cos\theta_{m}+F^{\prime }(\theta_{m})sin\theta_{m}\right)\\ +(F(\theta_{n})sin\theta_{n}-F(\theta_{m})sin\theta_{m})(F(\theta_{m})sin\theta_{m}-F^{\prime }(\theta_{m})cos\theta_{m}) \end{array}\right\}\\ \cdot \frac{1}{\sqrt{F^{2}(\theta_{m})+F^{\prime 2}(\theta_{m})}}H_{1}^{\left(2\right)}(k_{0}\rho )\sqrt{F^{2}(\theta_{n})+F^{\prime 2}(\theta_{n})}∆\theta \end{array} & ,\;\;m\neq n\\ \frac{1}{2} & ,\;\;m=n \end{array}\right.$$

Similarly, take the dot product of Equation (6) with $V_{2m}=\delta \left(x-x_{m}\right)\;\delta \left(y-y_{m}\right)$.

(17) $$\left\langle H_{z}^{s}\left(\overset{⃑}{r}\right),V_{2m}\right\rangle =\sum_{n=1}^{N_{d}}{\left(G_{2}\right)}_{mn}{\left(J_{sm}\right)}_{n}$$

where

(18) $${\left(G_{2}\right)}_{mn}=\frac{\omega \varepsilon_{0}}{4}∆c_{n}H_{0}^{\left(2\right)}\left[k_{0}\sqrt{{\left(x_{m}-F(\theta_{n})cos\theta_{n}\right)}^{2}+{\left(y_{m}-F(\theta_{n})sin\theta_{n}\right)}^{2}}\;\right]$$

**Figure 2:**
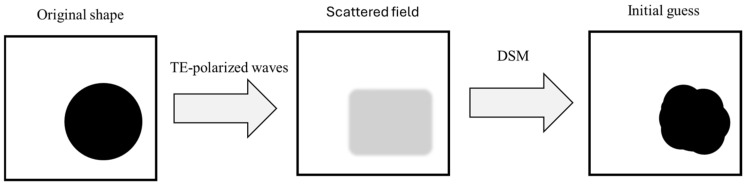
DSM schematic diagram.

The DSM is a straightforward, efficient, and non-iterative technique designed to retrieve the shape and location of an unknown scatterer. Its schematic diagram is shown in Figure 2. Originally developed for two-dimensional dielectric inverse scattering problems under fixed-plane wave illumination, the DSM has been extended to address a broader range of inverse scattering applications. Its efficiency is primarily attributed to the fact that it bypasses complex operations like singular value decomposition and the resolution of ill-posed integral equations. Furthermore, the DSM demonstrates strong robustness against random noise, making it a reliable approach for inverse scattering problems. In this study, for reconstructing surface and conductor shapes, the DSM demonstrates particular effectiveness in identifying the geometry and position of unknown scattering objects, as conductors only require shape and size reconstruction. The indicator function used in the DSM is formulated as follows:

(19) $$\Psi \left(r_{p}\right)=\frac{1}{N_{i}}\sum_{l=1}^{N_{i}}\frac{\left|\langle \bar{H_{l}^{s}}\left(r\right),\bar{G_{2}}(r,r_{p})\rangle \right|}{\left\|\bar{H_{l}^{s}}\left(r\right)\right\|\left\|\bar{G_{2}}(r,r_{p})\right\|}$$

where $r_{p}$ denotes a single sampling point, and r represents the receiver position with a total of $N_{r}$ receiver points. $N_{i}$ denotes the number of transmitters. $\bar{H_{l}^{s}}\left(r\right)$ is the vector of $N_{r}$ × 1 and $\bar{G_{2}}(r,r_{p})$ is the $N_{r}$ × 1 vector. If $\Psi \left(r_{p}\right)$ ≈ 1, then the sampling point $r_{p}$ is considered to lie within the unknown scatterer. And, if $\Psi \left(r_{p}\right)$ ≈ 0, the sampling point $r_{p}$ is located in the background medium.

## 3. U-Net

**Figure 3:**
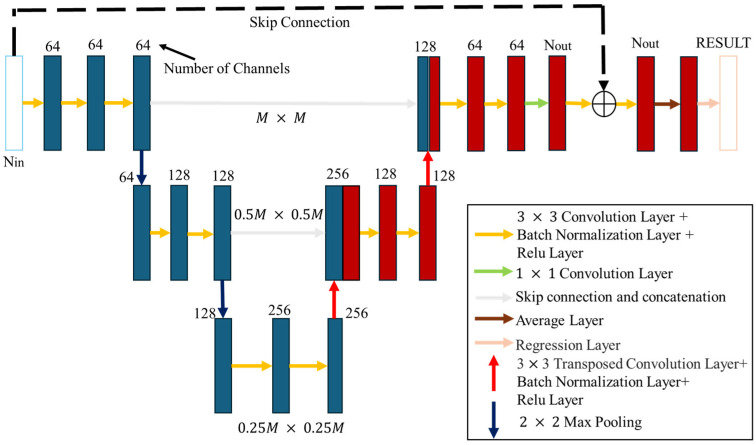
U-Net architecture. The black dashed arrow denote skip connect.

The U-Net architecture is composed of a contracting network on the left side and an expanding network on the right side, as illustrated in Figure 3. This architecture includes repeated application of 3×3 convolutional layers, batch normalization layers, and Rectified Linear Unit (ReLU) layers. In the pooling layers of the contracting network, a 2×2  max-pooling layer is employed, whereas in the pooling layers of the expanding network, a 3×3 transposed convolution layer is utilized.

The convolutional layer is mainly tasked with feature detection from the input image. The process involves transforming the input image into a matrix format, followed by the application of multiple convolutional filters., which slide over the matrix to produce feature maps. Critical attributes of the image, including spatial size and stride, are captured within the generated feature maps. Batch normalization layers help reduce training time by reducing the sensitivity of gradients to parameter initialization, thus enhancing the network’s overall accuracy. The ReLU activation function introduces nonlinearity, improving the network’s capacity to recognize intricate patterns and improve predictive accuracy. However, while such corrections contribute to enhanced training performance, the impact may not be readily observable.

Pooling layers are designed to reduce the dimensionality of feature maps, therefore lowering the number of parameters that require learning, which also helps mitigate overfitting. Maximum values are selected from separate regions of the feature map, with care taken to prevent overlap between selected areas. In the contracting network, pooling is used for down-sampling, while in the expanding network, it is used for up-sampling, ensuring that the quantity of input channels $N_{i}$ matches the quantity of output channels $N_{out}$.

In the final stage, to complete the network architecture, a 1 × 1 convolutional layer is incorporated, effectively acting as a fully connected layer responsible for final decision-making, which integrates the extracted features and generates the predictions [19]. The minimization equation is given as follows:

(20) argminAi,i:∑N=1NtfAi1Fα,F +Qi

Here, $A_{i}$ refers to the neural network architecture and its corresponding parameters, f denotes the error function, $F^{\alpha }$ represents the approximated conductor shape, and $Q\left(i\right)$ denotes the regularization function.

One of the major advantages of U-Net is that it uses “skip connections” to connect corresponding layers in the contracting and expanding paths. These connections can restore the spatial information lost during the down-sampling process, thereby enhancing reconstruction accuracy, especially for better recognition of fine structures. In addition, U-Net demonstrates high data efficiency, making it suitable for problems with limited training data, as is often the case in electromagnetic imaging applications. Its modular design is also highly flexible and can be applied to multi-category image segmentation, 3D reconstruction, and even integration of multimodal image data, rendering it a powerful tool for solving a variety of problems. In real-world practice, U-Net has demonstrated high robustness and good generalization capabilities in multiple areas like biomedical imaging, industrial detection, and remote sensing, effectively showcasing its versatility and value.

**Figure 4:**
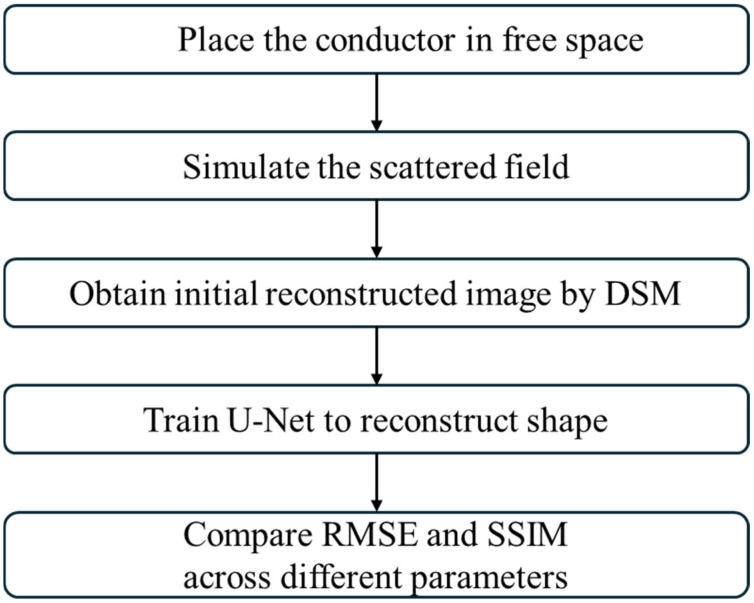
Flowchart of the experimental process.

The complete experimental flowchart is shown in Figure 4. The procedure begins by placing the conductors in free space. Next, the conductors are illuminated by a TE-polarized wave. The resulting scattered field data from both training and test sets are then input into the DSM for initial reconstruction. The dataset is fed into the U-Net training for image reconstruction. The Root Mean Square Error (RMSE) and Structural Similarity Index Measure (SSIM) are compared in different level noise conditions.

## 4. Numerical Results

This section presents a simulation setup that is established to recreate a conductor in free space. Repeated illumination with TE-polarized waves enables the collection of complete scattered field data.

A TE-polarized incident wave with a frequency of 3 GHz is employed to illuminate the conductors. A total of 64 transmitters and receivers are uniformly distributed around the scatterers at angular intervals of 5.625 degrees. The Domain of Interest (DOI) spans an area of 0.6 m×0.6 m, which is discretized into a 64×64 pixel grid. Conductors of different shapes are placed at random positions in the DOI.

We select Adaptive Moment estimation (ADAM) in the training since it is usually used in practice. For the ADAM parameters, the learning rate is configured as $10^{-3}$ to $10^{-5}$ and halved at every 20 epochs. The batch size is 32, and the maximum number of epochs is 40. To measure the effectiveness of each scheme, we define the RMSE formula as follows:

(21) $$RMSE=\frac{1}{M}\sum_{i=1}^{M}{\left\|Y-Y^{i}\right\|}_{F_{r}}/{\left\|Y\right\|}_{F_{r}}$$

Here Y and $Y^{i}$ denote the original shape and the reconstructed shape, in that order, M represents the total number of test cases, and $F_{r}$ represents the Frobenius norm. In order to compare the reconstruction results of each graph trained by the neural-like network, we define the SSIM as follows:

(22) $$SSIM=\frac{\left(2\mu_{\tilde{y}}\mu_{y}+C_{1}\right)\left(2\sigma_{ỹy}+C_{2}\right)}{\left({\mu_{\tilde{y}}}^{2}+{\mu_{y}}^{2}+C_{1}\right)\left({\sigma_{\tilde{y}}}^{2}+{\sigma_{y}}^{2}+C_{2}\right)}$$

where $\tilde{y}$ and y are the reconstruction and original shapes, in that order. $\mu_{y}$ represents the average pixel value of y, ${\sigma_{y}}^{2}$ is the variance of y, and $\sigma_{\tilde{y}y}$ is the covariance of ỹ and y. $C_{1}$ and $C_{2}$ are constants introduced to stabilize the division in cases of weak denominators.

**Figure 5:**
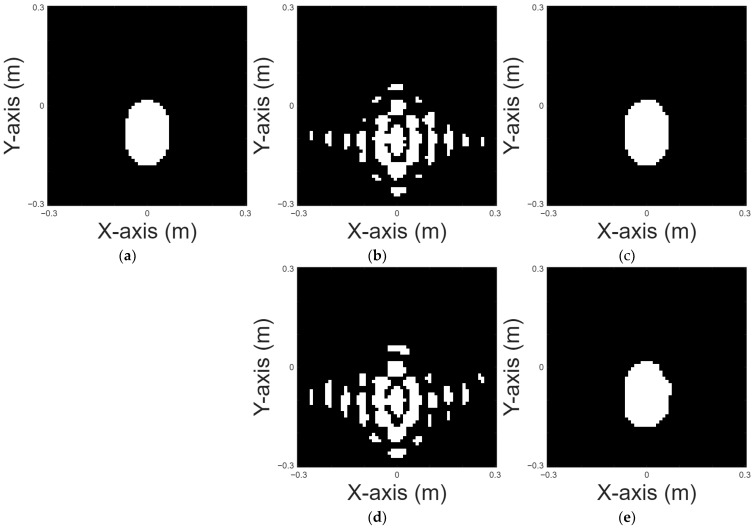
Reconstructed image of an oval shape: (**a**) true shape, (**b**) DSM with 10% noise, (**c**) U-Net with 10% noise, (**d**) DSM with 20% noise, and (**e**) U-Net with 20% noise.

An oval shape is chosen for case 1. Figure 5a displays the original shape of an oval shape. Figure 5b is the reconstructed image of an oval shape by the DSM with 10% noise and Figure 5c is the reconstructed image of an oval shape by U-Net with 10% noise. Figure 5d is the reconstructed image of an oval shape by the DSM with 20% noise and Figure 5e is the reconstructed image of an oval shape by U-Net with 20% noise. Figure 5b,d depict the reconstruction results obtained using the DSM for oval-shaped perfectly conducting objects under two different levels of additive noise. As shown, the DSM reconstructions suffer from shape deformation and noticeable background artifacts, particularly around the conductor boundaries. These deficiencies highlight the method’s limited robustness in handling noisy measurements. In contrast, Figure 5c,e present the corresponding reconstruction outcomes refined by the U-Net model. The results clearly demonstrate that the proposed deep learning framework effectively mitigates the noise-induced artifacts and recovers the conductor’s geometry with significantly improved accuracy and continuity. This comparison confirms the added value of incorporating neural networks to enhance the quality of initial estimates obtained by DSM. Table 1 presents the RMSE and SSIM results for the reconstruction by the DSM. Table 2 presents the RMSE and SSIM results for the reconstruction by the U-Net.

**Figure 6:**
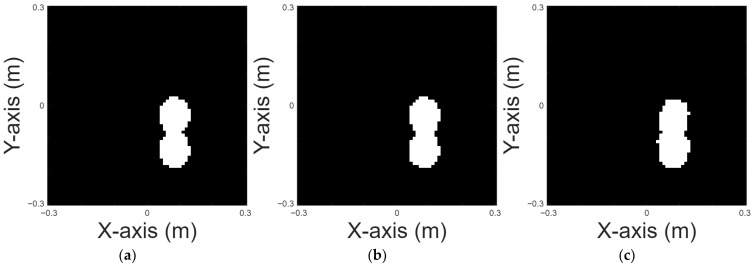
Reconstructed image of a peanut shape: (**a**) true shape, (**b**) with 10% noise, and (**c**) with 20% noise.

A peanut shape is chosen for case 2. Figure 6a displays the original shape. Figure 6b and 6c, respectively, show the images reconstructed by U-Net with 10% and 20% noise in the DSM training sets. Table 3 presents the corresponding RMSE and SSIM results for the reconstruction.

**Figure 7:**
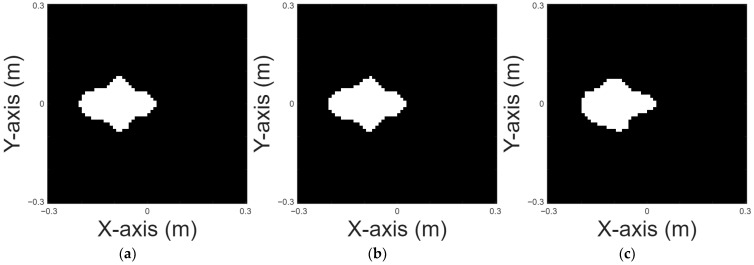
Reconstructed image of a UFO shape: (**a**) true shape, (**b**) with 10% noise, and (**c**) with 20% noise.

A UFO shape is chosen for case 3. Figure 7a displays the original shape; Figure 7b and 7c, respectively, show the images reconstructed by U-Net with 10% and 20% noise in the DSM training sets. Table 4 presents the RMSE and SSIM results for the reconstruction.

**Figure 8:**
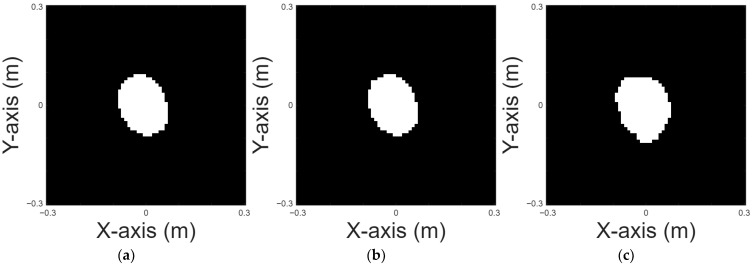
Reconstructed image of a pea: (**a**) True shape (**b**) with 10% noise (**c**) with 20% noise.

A pea shape is chosen for case 4. Figure 8a displays the original pea shape; Figure 8b and 8c, respectively, show the images reconstructed by U-Net with 10% and 20% noise in the DSM training sets. Table 5 presents the RMSE and SSIM results for the reconstruction.

**Figure 9:**
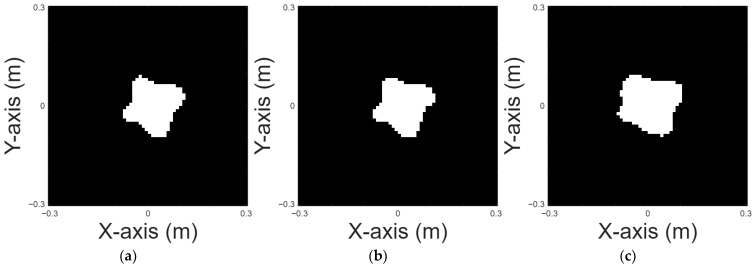
Reconstructed image of a four-petal shape: (**a**) true shape, (**b**) with 10% noise, and (**c**) with 20% noise.

Four-petal shape is chosen for case 5. Figure 9a displays the original shape; Figure 9b and 9c, respectively, show the images reconstructed by U-Net with 10% and 20% noise in the DSM training sets. Table 6 provides the RMSE and SSIM results for the reconstruction.

**Figure 10:**
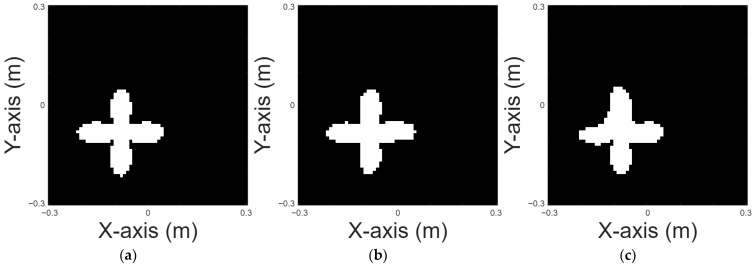
Reconstructed images of a cross shape: (**a**) true shape, (**b**) with 10% noise, and (**c**) with 20% noise.

A cross shape is chosen for case 6. Figure 10a displays the original shape; Figure 10b and Figure 10c, respectively, show the images reconstructed by U-Net with 10% and 20% noise in the DSM training sets. Table 7 provides the RMSE and SSIM results for the reconstruction.

**Figure 11:**
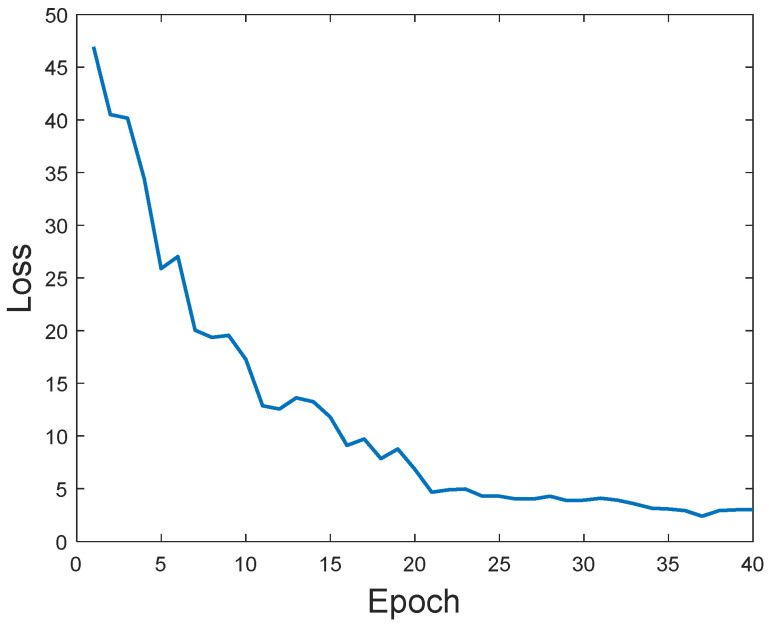
The training loss versus epochs.

Figure 11 shows the progression of the training process by displaying how the loss value decreases with the number of training epochs. The loss function quantifies the discrepancy between U-Net’s reconstructed image (shape) and the corresponding ground truth. In the first five training cycles, the loss drops sharply, indicating a rapid learning phase. Between the 5th and 20th cycles, the loss continues to decrease significantly, and between the 20th and 40th cycles, the downward trend becomes more gradual, suggesting that the network is approaching convergence.

## 5. Conclusions

In this study, we present the reconstruction capability for perfect conductors illuminated with TE-polarized waves using U-Net. The proposed method is structured as a five-stage pipeline: (1) TE-polarized waves illuminate the target conductor; (2) the scattered fields in the x- and y-directions are collected and used in forward modeling; (3) the Direct Sampling Method (DSM) produces an initial estimate of the shape; (4) a U-Net is trained with DSM-derived data to learn optimal parameters; and (5) the trained model is used to reconstruct high-resolution images. We derive the integral equation for the scattered field using the concept of induced currents and solve it using the method of moments. First, we apply the DSM to estimate the initial shapes of conductors. The results show that the DSM cannot clearly reconstruct the image, with an RMSE between 17% and 26%, and only indicates the relative position. Consequently, we adopt U-Net to achieve better reconstruction. Based on the results of our numerical simulations, under 10% noise and 20% noise, U-Net performs more clearly than the DSM, with an RMSE below 15%. Despite the high nonlinearity of the TE waves, AI technology has a significant effect and is timesaving for ISP. While practical TE wave imaging systems are still under development, our framework can be adapted to future implementations using mmWave arrays or terahertz sensing platforms. In future studies, we plan to integrate a Generative Adversarial Network (GAN) with advanced AI techniques. Additionally, we will strive to address more advanced scenarios like the buried space.

## Figures and Tables

**Table 1:** The RMSE and SSIM performance for an oval by the DSM.

	Noise Level	10%	20%
Performance			
RMSE		21.51%	22.70%
SSIM		66.87%	64.33%

**Table 2:** The RMSE and SSIM performance for an oval by the U-Net.

	Noise Level	10%	20%
Performance			
RMSE		2.49%	5.56%
SSIM		99.41%	97.11%

**Table 3:** The RMSE and SSIM performance for peanut.

	Noise Level	10%	20%
Performance			
RMSE		2.53%	7.18%
SSIM		99.52%	95.82%

**Table 4:** The RMSE and SSIM performance for UFO.

	Noise Level	10%	20%
Performance			
RMSE		3.51%	8.37%
SSIM		98.82%	94.07%

**Table 5:** The RMSE and SSIM performance for pea.

	Noise Level	10%	20%
Performance			
RMSE		2.03%	9.76%
SSIM		99.55%	92.03%

**Table 6:** The RMSE and SSIM performance for four-petal.

	Noise Level	10%	20%
Performance			
RMSE		2.49%	11.51%
SSIM		99.36%	90.68%

**Table 7:** The RMSE and SSIM performance for cross.

	Noise Level	10%	20%
Performance			
RMSE		6.81%	8.76%
SSIM		96.1%	94.23%

## Data Availability

Data are contained within the article.
